# In Memoriam

**Published:** 1889-01

**Authors:** 


### IN MEMORIAM.

On Sunday, the eleventh day of November, 1888, death suddenly removed from this earthly existence Dr. William R. Childs, one of the most faithful and highly esteemed members of this Board, and for many years its Secretary.

It therefore is fitting that the Medical Board of the Homœopathic Hospital of Pittsburg, Pennsylvania, give expression to the profound grief felt at this sudden taking off of one of our most valuable members—one of the most regular and punctual in his attendance at the meetings of the Board—nothing but sickness ever kept him from his place at the Secretary's desk; one of the most faithful and kind in his attendance on the sick and injured under his care in the hospital, and one of the most genial and friendly in all his intercourse with his fellow-members on this Board; and that we place on record a lasting testimonial of our regard for, and love of Dr. William R. Childs. Therefore

*Resolved*, That by his death the Medical Board of the Homœopathic Hospital has sustained a loss that words fail to express; that the surgical staff has lost one of its most skillful, careful, and successful operators; always punctual, exact, and methodical in every detail; gentle but firm, kind but impartial, true to the trust reposed in him; that the medical profession has lost a scholarly, dignified, and conscientious physician, and a brave, skillful, and successful surgeon; that his orphaned children have lost a kind, loving, and indulgent father; and that the community has lost a whole-souled, unselfish, genial man.

*Resolved*, That we tender to the bereaved family of Dr.

Childs, in this their hour of affliction, our sympathy and our condolence.

*Resolved*, That this testimonial be entered upon the records of the Medical Board of the Homœopathic Hospital of Pittsburg, and a copy thereof, suitably engrossed, be placed in the hands of the family of our late fellow-member; and also copies be sent to the medical journals of our School.

*Resolved*, That as a final tribute and token of love and respect, the Medical Board attend the funeral in a body.

> "He was a man, take him for all in all,
> We shall not look upon his like again."

W. J. Martin, M. D.,
M. J. Chapman, M. D.,
W. F. Edmondson, M. D.;
*Committee.*

